# Bone Diagenesis in Short Timescales: Insights from an Exploratory Proteomic Analysis

**DOI:** 10.3390/biology10060460

**Published:** 2021-05-23

**Authors:** Noemi Procopio, Caley A. Mein, Sefora Starace, Andrea Bonicelli, Anna Williams

**Affiliations:** 1 Forensic Science Research Group, Faculty of Health and Life Sciences, Applied Sciences, Northumbria University, Newcastle Upon Tyne NE1 8ST, UK; andrea.bonicelli@northumbria.ac.uk; 2 School of Applied Sciences, University of Huddersfield, Queensgate, Huddersfield HD1 3DH, UK; CAMein@uclan.ac.uk (C.A.M.); AWilliams34@uclan.ac.uk (A.W.); 3 School of Natural Sciences, University of Central Lancashire, Preston PR1 2HE, UK; 4 Dipartimento di Chimica, University of Turin, Via P. Giuria 7, 10125 Turin, Italy; sefora.starace@edu.unito.it

**Keywords:** taphonomy, bone proteomics, PMI, microbial decomposition, bioerosion, forensic sciences

## Abstract

**Simple Summary:**

Understanding the origin of bone degradation led by bacterial decomposition is essential in order to allow for the creation of better models to estimate the time elapsed since death for forensic casework, as well as for the preservation of archaeological specimens over the course of time. Within this study we applied modern proteomic technologies in order to evaluate how proteins in decomposing rat bones are affected by different post-mortem conditions, such as different depositional environments (buried versus exposed samples) and different sample types (whole carcasses versus fleshed limbs versus defleshed bones), over a period of 28 weeks. We found that the abundance of specific proteins was associated either with a microbial-led type of decomposition or with a specific post-depositional environment. Overall, this study shows that proteomic analyses can be useful to identify microbially- versus non-microbially driven decomposition, and that specific proteins—such as bone marrow and plasma proteins—can be more affected than others by extrinsic agents, whereas calcium-binding proteins seem to be more affected by microbial degradation.

**Abstract:**

The evaluation of bone diagenetic phenomena in archaeological timescales has a long history; however, little is known about the origins of the microbes driving bone diagenesis, nor about the extent of bone diagenesis in short timeframes—such as in forensic contexts. Previously, the analysis of non-collagenous proteins (NCPs) through bottom-up proteomics revealed the presence of potential biomarkers useful in estimating the post-mortem interval (PMI). However, there is still a great need for enhancing the understanding of the diagenetic processes taking place in forensic timeframes, and to clarify whether proteomic analyses can help to develop better models for estimating PMI reliably. To address these knowledge gaps, we designed an experiment based on whole rat carcasses, defleshed long rat bones, and excised but still-fleshed rat limbs, which were either buried in soil or exposed on a clean plastic surface, left to decompose for 28 weeks, and retrieved at different time intervals. This study aimed to assess differences in bone protein relative abundances for the various deposition modalities and intervals. We further evaluated the effects that extrinsic factors, autolysis, and gut and soil bacteria had on bone diagenesis via bottom-up proteomics. Results showed six proteins whose abundance was significantly different between samples subjected to either microbial decomposition (gut or soil bacteria) or to environmental factors. In particular, muscle- and calcium-binding proteins were found to be more prone to degradation by bacterial attack, whereas plasma and bone marrow proteins were more susceptible to exposure to extrinsic agents. Our results suggest that both gut and soil bacteria play key roles in bone diagenesis and protein decay in relatively short timescales, and that bone proteomics is a proficient resource with which to identify microbially-driven versus extrinsically-driven diagenesis.

## 1. Introduction

Bones are among the longest-preserved biological tissues in nature. Nevertheless, it is known that their preservation in both exposed conditions and buried contexts is affected by a multitude of extrinsic environmental variables, including physical and chemical environmental agents, scavengers, soil hydrology and pH, temperature, and microorganism-driven bioerosion [1]. Bone diagenetic processes have been extensively investigated over long timescales (e.g., in the archaeological records), with the aim of better understanding the taphonomic processes driving bones’ preservation and their ultimate conversion into fossil specimens [2,3,4], as well as the origin of the microbes driving bioerosion [5]. In particular, the effects of microbial attack and bone hydrolysis, resulting from intrinsic (i.e., derived from gut) or extrinsic (i.e., derived from soil and environment) microorganisms on the bone structure, are still a debated topic in archaeology and in palaeontology, and researchers have not yet fully agreed on which source can be considered to be the major driver for bone bioerosion [1,6]. In addition, these processes are less well understood when considering shorter timeframes (e.g., in forensic contexts), even though early taphonomy studies may benefit a wide range of fields, including forensic sciences, in addition to archaeology, biomolecular archaeology, and palaeontology.

Biomolecules in bones have been successfully found in a variety of archaeological and fossil records [7,8,9,10], as well as in forensic contexts (e.g., human bones collected from caseworks or cemeteries) [11,12], or in situations simulating forensic scenarios (e.g., using animal proxies to conduct bone proteomic studies) [13,14]. In particular, proteins have been shown to survive better than DNA [9,15]. Bone proteomics is a promising tool with which to evaluate chronological bone degradation through the evaluation of the survival of collagen [16] and of the decay of non-collagenous proteins (NCPs) [17,18,19]. Several studies show that bone collagen content can be halved in less than a thousand years [20], depending on burial conditions, with microbial attack having a strong impact on its persistence, particularly for bones that have been buried/located in relatively cold regions, such as northern Europe [21]. In neutral conditions, bone collagen is considered to be predominantly stable and insoluble [22]. On the one hand, collagen is protected by the strong linkage with the mineral matrix of the bone, and on the other hand, it protects the hydroxyapatite from dissolution [22]. Collagen deterioration through hydrolysis is a complex process mainly driven by bacterial collagenases, despite the fact that chemical (non-enzymatical) hydrolysis can also happen in situations involving the presence of high temperatures or extreme pHs [23]. Enzymatically-driven hydrolysis requires the access of collagenases to the collagen helix—a situation that occurs only when there is enough space among crystallites to allow the penetration of the enzymes [23], such as when the matrix has been partially dissolved by the presence of, for example, acid metabolites generated by the putrefaction of the soft tissues during the cadaveric decomposition [22]. Bacterial proteases are not able to function at acidic pHs, so the environment should become more alkaline/neutral to allow collagenases in bones to work [20].

Despite the great number of works aimed at understanding the mechanisms behind collagen survival within bones, less is known regarding the degradation and subsistence of NCPs [24]. Various studies on archaeological and palaeontological bones suggested that NCPs may survive better than collagen, due to their strong affinity to the mineral matrix of the bone [17,25,26]. However, there are limited experimental studies exploring the mechanisms and the quantitative degradation of NCPs in forensic timeframes. A work based on the burial of swine carcasses showed that the majority of changes in the proteome occur in the first four months of the PMI [13]. The study highlighted two different behaviours: proteins for which the relative abundance significantly decreased during the first months, to then become more stable (plasma proteins); and proteins showing some degree of stability in the initial stages of decomposition, followed by a drastic decrement (muscle proteins) [13]. These findings are also supported by a study by Creamer and Buck [27], who employed luminol to prove the time-dependent degradation of haemoglobin—a methodology that can be applied to classify skeletal human remains as forensic or archaeological. In addition to haemoglobin, transferrin has always attracted attention, as it has been considered a reliable indicator of PMI, although it is not directly extracted from bone tissue [28]. Despite its potential, its degradation was seen to be highly temperature-dependent [28] and, therefore, not the most suitable biomarker for long PMIs when temperature can fluctuate considerably. The degradation of the muscle proteins identified in bone samples was previously interpreted as a link to the complete decomposition of superficial soft tissues at prolonged PMIs [13]. This finding is also supported in the literature, where similar patterns were seen for muscle proteins extracted from muscular tissue after various PMIs [29,30].

In order to understand the phenomena involved in the degradation of proteins in bones, it is essential to illustrate the changes to which a corpse is subjected from its death, as well as the role of microbial communities—also referred to by certain authors as the necrobiome—which are among the main agents involved in post-mortem tissue decomposition [31]. The first late post-mortem change is the autolytic process, which takes place just after death and is caused by the breakdown of cellular membranes, which causes the release of hydrolytic enzymes able to digest the surrounding tissues. During this process, the body environment is rapidly converted from an aerobic to an anaerobic one, allowing anoxic bacteria from the gut (endogenous bacteria) to multiply and the body to enter the putrefactive stage, which generally starts 1 hour post-mortem and lasts for 48 h [6]. Throughout putrefaction, the second stage of late post-mortem changes, bacteria transmigrate from the gut to the rest of the body via the blood vessels in a matter of hours [32,33], reaching the bones within a day of death [22]. The activity of endogenous bacteria via reductive catalysis results in bloating of the corpse, due to the accumulation of gases [22] that would eventually lead to abdominal rupture and the exposure of internal tissues, offering an ideal environment for exogenous microbial communities present in the soil and in the surrounding air [34,35,36]. These exogenous communities proliferate due to the richness of the nutrients offered by the decomposing corpse, replacing the endogenous communities [37]. With the progression of decomposition, specific bacterial species are attracted in a quite predictable way, regardless of the type of burial environment. For this reason, the microbial succession in soil has been studied by several authors as a means of estimating the PMI [38,39].

Although the post-mortem microbial succession has attracted significant interest for PMI estimation, the origin of the bacteria responsible for bone diagenesis is still hugely debated [1,5,6]. A study conducted by Damann et al. [40] identified changes in the abundances of Firmicutes, Actinobacteria, Acidobacteria, and Proteobacteria within human ribs at various decomposition stages. In particular, they found a predominance of gut bacteria during the earlier decomposition stages (e.g., partially skeletonised remains), and of soil bacteria during the advanced decomposition stages (e.g., dry remains) [40]. There is a consensus in the current literature that microbes (both bacteria and fungi) can affect the internal microstructure of bones [1,41,42]. White and Booth [1] proposed gut bacteria as the main drivers of the bone diagenetic processes; Reiche et al. [43] suggested instead that soil microbes are mainly responsible for bone diagenesis in archaeological samples. However, given the depth of burials, and the reduced number of soil microbial communities found at the average burial depth in contrast with shallower burial depths [44], it may be possible that soil microbes alone are not uniquely responsible for the degree of diagenesis observed in the work of Reiche et al. [43]. Jans [22] seems to attribute more importance to endogenous bacteria as drivers for bone bioerosion, whereas, more recently, Turner-Walker [5] suggested that soil bacteria are essential for bone diagenesis, and stated that the “hypothesis that bacterial tunnelling arises from gut bacteria, although plausible, is unproven”.

One aspect that should never be neglected is the importance of the depositional environment, which can also lead to measurable physical and chemical changes to the microstructure of the bone, due to microbial interactions between the hard tissue and the surrounding environment [43,45]. This introduces a large number of factors that have to be considered when evaluating the decomposition and decay of a corpse; therefore, systematic experiments are necessary in order to limit these variables and isolate the ones of interest. Thus, ultimately, both the decomposition process [46,47] and the depositional environment [37,47,48] can have strong effects on the extent of bone diagenesis, which seems to occur at different rates in buried bones when compared with exposed ones [49].

The present work represents a preliminary study that aims to fill the knowledge gaps concerning early bone diagenesis in forensic timeframes through the innovative approach of bone proteomics. We consider here the existence of three main microbial groups: endogenous microbes (derived predominantly from the gut), aerobic environmental microbes (present in the air surrounding exposed carcasses), and soil microbes (exogenous bacteria in the burial environment). We focused specifically on the proteomic alterations observed in bones exposed predominantly to the combined action of autolysis coupled with the effects that endogenous and environmental bacteria, soil bacteria, or environmental bacteria alone can have on bodies, over short timescales. It should be noted that the presence of the “environmental bacteria” also implies the contemporaneous presence of other living species (e.g., insects) able to colonise the decomposing bodies. The deposition times ranged between 4 and 28 weeks, and the combination between different “sample types” (whole remains, excised fleshed limbs, and defleshed bones) and “depositional environments” (exposed on a clear plastic surface in an outdoor environment or buried in soil) allowed the evaluation of the effects that these different microbial groups had on the bone proteomes. The objective of this approach is to evaluate the presence of proteomic alterations over short/forensic timescales, and assess the effects that specific decomposers and environmental variables have on them. We were able to clarify the potential that bone proteomics has in the evaluation of early diagenetic processes, and to elucidate some of the mechanisms underlying the NCPs’ preservation within bones in specific conditions within short timescales.

## 2. Materials and Methods

### 2.1. Field Experiment and Sample Composition

Eleven black rats (*Rattus Rattus*) were purchased frozen from a reptile centre (Northampton Reptile Centre), operating in accordance with the Animal Welfare Act 2006, compliant with ethical research standard practices. We used animal models in order to test different post-mortem conditions with minimal interindividual variability. The animals were flash frozen within ~2 h after death, delivered frozen, and stored at −20 °C until the beginning of the study. Five rats (named “whole body” and “control” for the purpose of this paper) were not subjected to any dissection, so they were intact until the beginning of the experiment. Six rats were used to obtain either back limbs (named “fleshed limb”) or back limb bones (named “defleshed bone”). Prior to dissection, they were defrosted overnight at 4 °C. Although we are aware of the effects that freeze-thawing can have on intrinsic bacterial communities [50] and on soft tissues and cell structures [51], several studies have showed that bacteria are able to survive during freezing procedures [52,53], so we are confident that this experimental choice has not impaired the correct evaluation of the decomposition phenomena associated with the presence of intrinsic bacteria in this study.

The experimental samples were buried at the University of Huddersfield’s animal taphonomic facility (HuddersFIELD), from mid-November 2018 to May 2019. The facility is situated on grassy farmland in West Yorkshire (UK). Local temperature and rainfall information was collected using a local weather station, World Weather Online, and can be found at (https://www.worldweatheronline.com/halifax-weatheraverages/west-yorkshire/gb.aspx, accessed on 1 November 2019). Average monthly temperature and rainfall readings/values were recorded from November 2018 to May 2019—the duration of the field experiments (Supplementary Materials, Figure S1).

The samples were deposited in either exposed or buried conditions. The exposed samples were placed into two large plastic boxes, one box for each tissue type (e.g., one box for the whole rats, one for the excised fleshed limbs). The boxes were open, had holes drilled in the base to allow drainage of rainwater, and were placed within large, locked wire cages in order to protect them from large scavengers. The buried samples were placed into smaller, soil-filled 3-L boxes, in order to prevent disturbance of the samples by scavengers. The samples were placed on top of ~1.35 L of Godwins topsoil and further covered with 1.35 L of the same topsoil. These boxes were closed and their lids were weighted down to protect the contents from scavengers. Upon collection, the defleshed bones were cleaned of any soil or debris using distilled water. The whole remains and the excised fleshed limbs were defleshed where necessary using a #10 scalpel. All bones were cleaned using distilled water and then stored in the freezer until further preparation. Each bone specimen was placed into a small clean polythene bag and gently broken into smaller fragments with a mortar. Three small fragments (sized ~3–4 mm^2^) were used as biological replicates (named “A”, “B”, and “C”) for each of the samples used (Table 1). As a result, we totalled 39 samples that were subjected to further proteomic extraction and analysis.

### 2.2. Protein Extraction

Proteins were extracted following the protocol proposed by Procopio and Buckley [54]. Briefly, each bone fragment was placed in a separated 1.5-mL tube and decalcified with 1 mL of 10% formic acid (Fisher Scientific, Loughborough, UK) for 6 hours at 4 °C. The acid-soluble fraction was discarded, and the pellet was then treated with 6 M guanidine hydrochloride (Sigma-Aldrich, Gillingham, UK)/100 mM TRIS buffer (Thermo Fisher Scientific, Paisley, UK) with an adjusted pH of 7.4 for 18 h at 4 °C. The acid-insoluble fraction was then exchanged with 50 mM ammonium acetate (Scientific Laboratories Supplies, Nottingham, UK) using 10 kDa molecular weight cut-off filters (Vivaspin^®^500 from Sartorius, Göttingen, Germany), and the proteins were then reduced with 5 mM dithiothreitol (Fluorochem, Hadfield, UK) for 40 min at room temperature, alkylated using 15 mM iodoacetamide (Sigma-Aldrich, UK) for 45 min in the dark at room temperature, and quenched by adding 5 mM dithiothreitol. Proteins were digested using trypsin (Promega, Southampton, UK) for 5 hours at 37 °C, and then frozen. We added 1 *v*/*v*% trifluoroacetic acid (Fluorochem, Hadfield, UK) to the samples prior to their desalting and purification, in order to bring them to a final concentration of 0.1 *v*/*v*% trifluoroacetic acid. The purification was achieved using OMIX C18 pipette tips (Agilent Technologies, Stockport, UK), in accordance with the manufacturer’s instructions. The peptides were finally eluted into 100 µL of 50 *v*/*v*% acetonitrile (Thermo Fisher Scientific, UK)/0.1 *v*/*v*% trifluoroacetic acid. Samples were then left in the fume cupboard with lids open in order to dry them completely at room temperature prior to their submission for LC–MS/MS analysis.

### 2.3. LC–MS/MS Analysis

Samples resuspended in 5 *v*/*v*% ACN/0.1 *v*/*v*% TFA were analysed via LC–MS/MS using an Ultimate™ 3000 Rapid Separation LC (RSLC) nano LC system (Dionex Corporation, Sunnyvale, CA, USA) coupled to a Q Exactive™ Plus Hybrid Quadrupole-Orbitrap Mass Spectrometer (Thermo Fisher Scientific, Waltham, MA, USA). Peptides were separated on an EASY-Spray™ reverse phase LC Column (500 mm × 75 µm diameter (i.d.), 2 µm, Thermo Fisher Scientific, Waltham, MA, USA) using a gradient from 96 *v*/*v*% A (0.1 *v*/*v*% FA in 5 *v*/*v*% ACN) and 4 *v*/*v*% B (0.1 *v*/*v*% FA in 95 *v*/*v*% ACN) to 8 *v*/*v*%, 30 *v*/*v*%, and 50% B at 14, 50, and 60 min, respectively, at a flow rate of 300 nL min^−1^. Acclaim™ PepMap™ 100 C18 LC Column (5 mm × 0.3 mm i.d., 5 µm, 100 Å, Thermo Fisher Scientific) was used as trap column at a flow rate of 25 µL min-1 kept at 45 °C. The LC separation was followed by a cleaning cycle with an additional 15 min of column equilibration time; then, peptide ions were analysed in full-scan MS scanning mode at 35,000 MS resolution with an automatic gain control (AGC) of 1e6, injection time of 200 ms, and scan range of 375–1400 m/z. The top 10 most abundant ions were selected for data-dependent MS/MS analysis with a normalized collision energy (NCE) level of 30, performed at 17,500 MS resolution with an AGC of 1 × 10^5^ and maximum injection time of 100 ms. The isolation window was set to 2.0 m/z, with an underfilled ratio of 0.4%, and dynamic exclusion was employed; thus, one repeat scan (i.e., two MS/MS scans in total) was acquired in a 45 s repeat duration, with the precursor being excluded for the subsequent 45 s.

### 2.4. Protein Identification and Statistical Analysis

Peptide mass spectra were then searched against the SWISS-PROT database using the Mascot search engine (version 2.5.1; www.matrixscience.com, accessed on 10 December 2019) for matches to primary protein sequences, specifying the taxonomy filter as *Rattus rattus*. This search included the fixed carbamidomethyl modification of cysteine, which results from the addition of DTT to proteins. In the light of our aims, deamidation (asparagine and glutamine) and oxidation (lysine, methionine and proline) were also considered as variable modifications. The enzyme was set to trypsin, with a maximum of two missed cleavages allowed. It was assumed that all spectra held either 2+ or 3+ charged precursors.

Progenesis QI for Proteomics software (version 4.2; Nonlinear Dynamics, Newcastle, UK) was used to identify the proteins and assess their relative abundance in each sample. The relative abundance of the proteins present in the samples is calculated by the software by measuring the peptide ion abundances as a result of the sum of the areas under the curve (AUC) for each peptide ion. The software normalizes each LC–MS/MS run against a reference run automatically selected as the normalization reference, in order to consider and to correct the systematic experimental variations that can occur between different runs; then, it enables protein comparisons between different experimental conditions, and the identification of protein expression changes. Peptide ions with a score of <23, indicating identity or extensive homology (*p* < 0.05), were excluded from the analysis based on the Mascot evaluation of the peptide score distribution. To further increase the reliability of the obtained results, proteins with a peptide count of <2 were also excluded from further analysis.

STRING software (version 11.0) was used to visualize links among the identified proteins, and to evaluate the significance of their interactions [55]. The confidence score set for showing interactions was set to “medium = 0.400”.

Statistical analysis was carried out on arcsinh normalised data [56], in the same way in which the Progenesis software operates. Principal component analysis (PCA) was performed on the normalized abundance values exported from Progenesis, using R software with the factoextra and ggplot2 packages, using proteins sorted by their ANOVA FDR adjusted *p* values (or “*q* value”) in order to exclude the proteins that showed similar relative abundances across different conditions (and that did not contribute to cluster separations). Protein abundances were considered to have significantly contributed to the explanation of the variance between different groups of samples when their ANOVA *p* < 0.05. For post-hoc analysis, Tukey’s HSD test was employed with significance set at *p* < 0.05 for pairwise comparison.

## 3. Results

**Figure 1:**
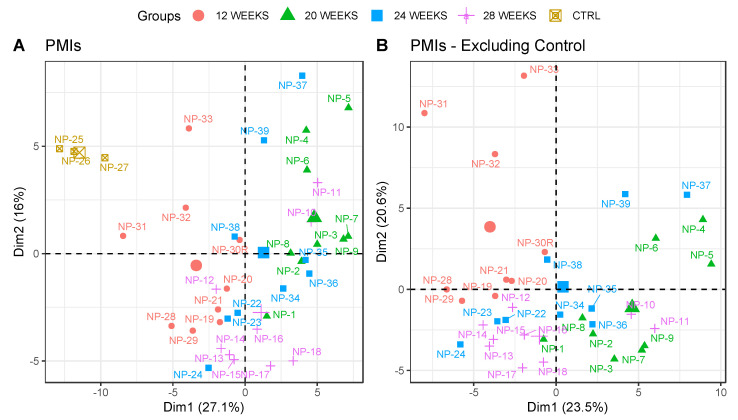
PCA plot representing the samples grouped by their PMIs; (**A**) including control samples, and (**B**) excluding control samples. The same-coloured icons indicate the same PMI, as reported in the legend. Control (CTRL) = 0 weeks PMI. Protein abundances used for the PCAs were the ones whose *q* values were significant between the different conditions (*n* = 90, *q* < 0.05). The bigger icons in the plot for each group represent the centroids of the samples in the group.

Overall, 67,490 MS/MS spectra were acquired from the LC–MS/MS analyses, and were searched against the SWISS-PROT database using Mascot. After the refinement steps previously mentioned, 2,539 search results (ions) were matched, and after the exclusion of proteins matched with less than 2 unique peptides, we obtained 168 proteins. In order to exclude the possibility that the proteomic results were associated with differences in the PMIs of the specimens, we grouped samples exclusively based on their PMIs, and plotted them on a PCA graph (Figure 1A). The samples did not cluster in clearly and defined positions, apart from the control samples, and the variability explained by summing the first and second components was less than 50%. To verify that the clustering was not affected by the inclusion of the control group in the model, we excluded those from the PCA (Figure 1B). Moreover, in this case, no clear clusters were identified, and samples belonging to different PMIs overlapped on both dimensions without any clear trend.

**Figure 2:**
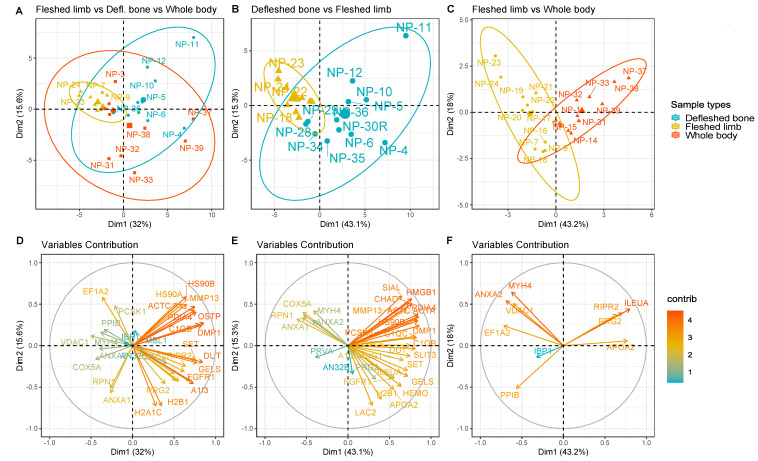
PCA plots (top) and variable maps (bottom) representing the samples grouped respectively in: (**A**,**D**) buried defleshed bones vs. exposed fleshed limbs; (**B**,**E**) exposed fleshed limbs vs. exposed whole bodies; and (**C**,**F**) buried defleshed bones vs. exposed fleshed limbs vs. exposed whole bodies. In each cluster, a bigger coordinate with the same colour was calculated as the centroids of the samples in the group. The same-coloured icons indicate the same condition. The ellipse shows a cluster categorised by the same condition (confidence interval of 0.95). Protein abundances used for the PCAs were the ones that were statistically significant according to their *q* values.

Samples were then grouped based on the depositional environment and on the sample type, in order to test whether or not the bone proteome was affected by it and, consequently, whether any variation in bone diagenesis could be observed via the analysis of the bone proteome. Results showed a significant difference in the proteomes of the three distinct sample types, and it was possible to identify specific proteins responsible for the variance observed, particularly when comparing the exposed fleshed samples with either the buried defleshed bones or the exposed whole bodies (Figure 2). The only situation in which it was not possible to identify proteins with significant *q* values was the exposed whole bodies versus the buried defleshed bones, meaning that the two groups shared a similar proteomic profile. The reported PCAs were able to cluster both fleshed and defleshed limbs (Figure 2B), and fleshed limbs versus whole bodies (Figure 2C), with models able to explain 58.4% and 61.2%, respectively, of the total variance between the two groups. We were also able to find the specific proteins that contributed the most to the variance observed in the PCAs (Figure 2E,F; red arrows).

**Figure 3:**
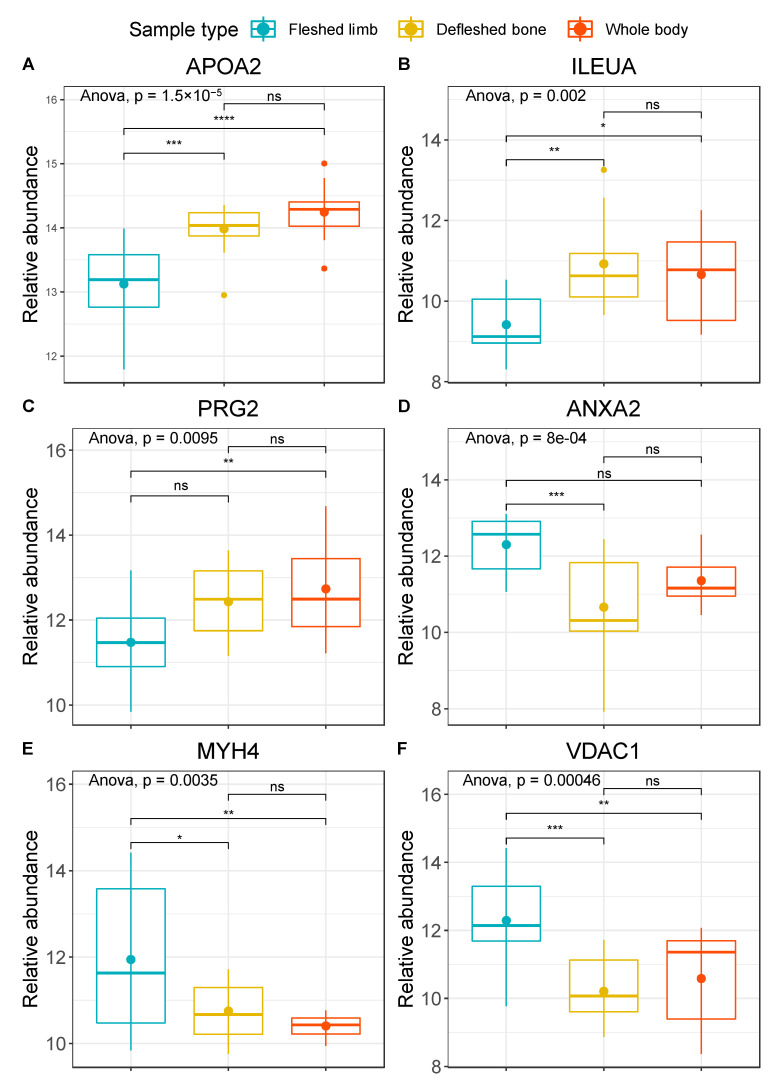
Boxplots with the relative abundances (arcsinh normalised) of (**A**) APOA2, (**B**) ILEUA, (**C**) PRG2, (**D**) ANXA2, (**E**) MYH4, and (**F**) VDAC1. Fleshed exposed limbs are represented in blue, defleshed buried bones in yellow, and exposed whole bodies in red. The plots provide extended *p* values for multivariate ANOVA and coded significance for Tukey’s HSD test (*p* < 0.0001 ‘****’; *p* < 0.001 ‘***’, *p* < 0.01 ‘**’, *p* < 0.05 ‘*’; *p* > 0.05 ‘ns’).

**Figure 4:**
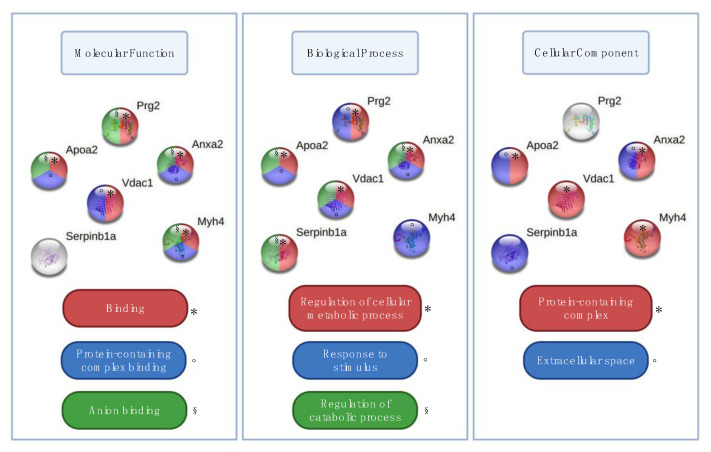
Functional enrichments in GO terms (molecular functions, biological processes, and cellular components) for the six proteins identified as being significant among the groups. For each GO class, different colours and symbols represent a specific GO term.

Starting from the significant contributors that were able to discriminate different groups of samples, we selected the ones that were significant (*q* < 0.05) both for fleshed limbs versus defleshed bones and for fleshed limbs versus whole bodies. We obtained six proteins in this manner, which differed in abundance when tested by post-hoc pairwise comparisons: apolipoprotein A-II (APOA2), leukocyte elastase inhibitor A (ILEUA), bone marrow proteoglycan (PRG2), annexin A2 (ANXA2), voltage-dependent anion-selective channel protein 1 (VDAC1), and myosin-4 (MYH4) (Figure 3). APOA2 is a plasma protein produced by the liver and commonly found in bone tissue [57]; ILEUA and PRG2 are bone marrow proteins [58,59]; ANXA2 is a bone specific protein associated with osteoclasts and bone tissue formation [60]; and MYH4 and VDAC1 are both skeletal muscle proteins [61,62]. When uploaded on the STRING software, these six proteins did not show significantly more interactions then expected, as predictable from the very small set of proteins (*n* = 6). However, according to Gene Ontology (GO) terms, we found functional enrichments for the molecular functions “binding” (*n* = 5; strength 0.56), “protein-containing complex binding” (*n* = 4; strength 1.45), and “anion binding” (*n* = 4; strength 1.04), for the biological processes “regulation of cellular metabolic process” (*n* = 5; strength 0.93), “response to stimulus” (*n* = 5; strength 0.72), and “regulation of catabolic process” (*n* = 4; strength 1.72), as well as for the cellular components “protein-containing complex” (*n* = 4; strength 0.859) and “extracellular space” (*n* = 3; strength 1.26) (Figure 4).

When examining the pairwise relationships between the different groups, it was clear that fleshed limbs differ from the other two groups. In particular, the abundance of APOA2 (Figure 3A) seemed to be significantly lower in fleshed limbs compared to both defleshed bones and whole bodies. These latter two groups did not show any significant differences between one another. ILEUA2 and PRG2 were also less abundant in fleshed limbs than in the other two sample types, and showed significant differences between fleshed limbs and both defleshed bones and whole bodies (Figure 3B), and between fleshed limbs and whole bodies (Figure 3C). In contrast, ANXA2 and VDAC1 were more abundant in limbs covered in flesh compared to the remaining groups, and the differences were moderately-to-highly significant (Figure 3D,F). Finally, MYH4 showed a significant difference between fleshed limb and whole-body deposition, and a less strong (but still significant) difference between fleshed limbs and defleshed bones (Figure 3E). The same trend also applies when the different type of depositional environment is taken into consideration, as defleshed bones were buried, whereas fleshed limbs and whole bodies were placed on the plastic surface and exposed to environmental factors (such as humidity, insects, environmental microorganisms, etc.).

## 4. Discussion

This work was aimed at understanding whether significant differences could be found among the proteomes extracted from limb bones deposited/buried into different depositional environments and subjected to different post-mortem conditions, which should have involved different causative agents and impacted the progression of bioerosion and early diagenetic phenomena, over a relatively short period of time ranging from 0 to 28 weeks. In order to do so, we examined the proteomes collected from either exposed whole remains, exposed fleshed back limbs, or buried defleshed bones.

To evaluate which parameters might have contributed the most to the observed proteomic results and associated diagenetic phenomena, it is important to summarize which variables were expected to take place in decomposition in the various sample types and depositional environments tested. Whole bodies exposed to the surface were subjected to the effects of autolysis and to the combined action of both intrinsic bacteria and environmental aerobic bacteria and insects (e.g., blowflies). In this case, the environmental conditions (e.g., temperature, humidity, rainfall, etc.) have also directly impacted the decomposition rate of the carcasses. A very similar situation can be expected for the fleshed limbs exposed on the surface, with the main difference being that limbs disarticulated from the rest of the body may or may not have been colonised by gut bacteria, depending on the speed at which those bacteria travelled along the vascular system reaching the limbs (and consequently the bones) post-mortem and prior to their dissection. Hence, overall, the effects that internal bacteria had on bone diagenesis and bioerosion should be limited in comparison with those observed on whole carcasses. Finally, the last scenario is the one that differs the most from the other two; in this last case, buried defleshed bones were subjected predominantly to the action of exogenous (e.g., soil) bacteria, soil insects, and autolytic phenomena, and only in a minor way by the presence of intrinsic gut bacteria that eventually managed to reach the bones prior to their disarticulation. Moreover, the environmental conditions affected the decomposition of these samples in a less direct way, due to the surrounding soil matrix that protected them from, for example, drastic temperature changes, direct rainfall, and changes in humidity.

Within this work we did not find significant differences in relation to specific PMIs, but we observed a clear difference between the proteomes of the exposed fleshed limbs and those of the exposed whole remains or buried defleshed bones. In particular, we highlighted six proteins whose abundance was significantly affected by the sample type and by the environmental deposition.

Between the proteomes from the exposed fleshed limbs (subjected predominantly to autolysis and to environmental microorganisms and factors) and from the buried defleshed bones (subjected predominantly to the action of autolysis and soil bacteria), we found APOA2 to be the greatest contributor among the six proteins with different abundances within the various conditions tested. APOA2 is a plasma protein, and was seen to be significantly less abundant in fleshed exposed limbs compared to the other two groups (Figure 3A). No significant differences were shown between buried bones and whole bodies. This suggests that the presence of a cut on the limb (e.g., following the excision of the limb from the body) and the following exposure of the tissues to the external environment allows the bodily fluids (including blood) to flow out faster compared with the whole carcasses. Additionally, bones exposed to external factors (including insects and bacteria, but also environmental conditions) can be subjected to considerably more protein decay than bones either “protected” by an intact carcass or surrounded by soil. It is frequent to find plasma and muscle proteins in bones as residuals from surrounding soft tissues, even when muscles are carefully removed (e.g., in the example of defleshed bones, Procopio and Buckley, data not published). Overall, this could explain the decrease in the number of plasma proteins found in specimens taken from the fleshed limbs group.

These possible interpretations are supported by the fact that the carcasses did not reach the complete skeletonization stage (according to their total body scores in accordance with the table proposed by Adlam and Simmons [63], data not shown) at the end of the experiment, with bone exposure being only <50% of the scored area.

We also noticed similar behaviour for two bone marrow proteins—namely, ILEUA and PRG2—although their contribution to the observed variance was smaller than that found for APOA2. These bone marrow proteins showed similar abundances in the buried bones and in the whole-body samples, but were notably less abundant in the exposed fleshed limbs. This further supports our interpretation that the burial environment and the entire body mass could function as protection from external environmental conditions, reducing the time of exposure of the bone to the mechanical action of rain. These results indicate that bone marrow and plasma proteins are degraded in a faster way in the exposed fleshed limbs in comparison with exposed whole bodies and buried bones, although the reasons for this finding are still not entirely clear, and warrant further investigation. It does not seem illogical that this behaviour is linked to groups of proteins with a lower affinity for hydroxyapatite and bone collagen [17]. In the theoretical absence of soil or gut bacteria, proteins of these groups are mostly degraded by autolysis and bioerosion, while mineral-binding proteins have shown more longevity and, therefore, represent an ideal target for archaeological research [4,24,46].

MYH4, ANXA2, and VDAC1 were found to be more abundant in the fleshed samples than in the whole ones or in the buried bones, indicating that these proteins may have been attacked by bacteria (either gut or soil bacteria) more than by the extrinsic factors. ANXA2 is a calcium-binding protein involved in osteoclast formation and bone resorption [64]. The higher abundance found in the exposed fleshed limbs could reflect the fact that proteins more intimately connected to hydroxyapatite are less prone to degradation from autolytic processes and environmental factors, but are attacked by the microbial action during bone diagenesis, even in relatively short forensic time frames. Again, for this protein significant differences were only found between fleshed limbs and the remaining two groups, but not between defleshed bones and whole bodies. These findings support what was previously stated for plasma and marrow proteins. The presence of both gut and soil bacteria here plays a major role in reducing the abundance of proteins with high calcium ion affinity. Despite no significance difference being found between the two groups, and so between the two distinct bacterial sources, Figure 3D shows the detrimental effect of microbial action on this protein.

MYH4 and VDAC1 behaved in a similar way to ANXA2. Both of these proteins are highly expressed in the skeletal muscle: myosins are well known for their ATPase activity in the skeletal muscle that allows muscle contraction [65], while VDAC1 is involved in the transport of ATP in the sarcoplasmic reticulum of the skeletal muscles (in addition to mitochondria) [66]. The abundance of these proteins was constantly reduced in the presence of either gut or soil bacteria, as shown by the very small deviations from the median recorded for the defleshed bones and for the whole-body samples. The identification of muscle proteins through bone proteomic analyses is not new or surprising, and has been previously shown where the PMI was not long enough to allow for the complete decomposition of the soft tissues and of the proteins associated with them [13,14]. The fact that muscle proteins were degraded more effectively in the samples where gut bacteria were present (e.g., whole bodies) than in the exposed limbs was expected, since the autolytic processes on muscle tissue normally take place in the first 24–28 h post-mortem [67], and after this period, gut bacteria are the main drivers of additional tissue degradation. Additionally, the low abundance of muscle proteins in buried bones can be explained by the action of soil bacteria combined with the reduced amount of skeletal muscle tissue available at the starting point of the decomposition (after defleshing).

Despite the promising results, the present study is not without limitations. One of these is the use of rats instead of the more commonly used pigs as analogues for humans in decomposition studies. However, it is not uncommon to find forensic studies where small animals (such as rats or mice) have been used instead of pigs in order to increase the sample size and the reproducibility of the results among biological replicates (e.g., same breed, age, food intake, etc.) [38]. Additionally, to further improve the understanding of bone taphonomic phenomena and bioerosion via proteomics, future studies should include more frequent timepoints for sample collection, and could be expanded to prolonged timescales, with an increased number of samples and with the inclusion of an additional indoor/laboratory depositional environment that will exclude the presence of environmental bacteria in order to simplify the model and the interpretation of the results. This will eventually allow for a better interpretation of the interactions between different factors and bone proteomes by implementing more explanatory statistical analyses.

## 5. Conclusions

Overall, these findings suggest that, despite the fact that bone proteomics does not allow for the distinction between protein degradation caused by different sources of bacteria—such as gut or soil bacteria—it does allow for the discrimination of samples subjected either to bioerosion or to the action of extrinsic factors. This study allowed us to understand how and which NCPs are more degraded in different scenarios, ultimately providing insights into the survival of bone biomolecules within different conditions. More specifically, the results reported in the present study show that muscle proteins and calcium- and collagen-binding proteins are more prone to degradation by bacterial attack than by hydrolytic and extrinsic processes, even after relatively short timeframes, such as the ones investigated in our study. On the other hand, plasma and bone marrow proteins seem to be protected by the presence of an intact body mass or by the burial environment. To conclude, proteomic analyses show the potential to reveal information that cannot be obtained with more classical approaches regarding taphonomic events occurring in relatively short timeframes, and this should be considered for future studies aimed at better understanding the extent of diagenesis in various conditions, and of the decay of the biomolecules in bones.

## Figures and Tables

**Table 1:** Samples used for proteomics, with the sample codes, depositional environment, sample type, post-mortem timescale, and proteomics code associated with them.

Sample Codes	Depositional Environment	Sample Type	Timescale in Weeks	Proteomics Code
CTRL A-B-C	Control	Control	0	NP25-26-27
w12 BB A-B-C	Buried	Defleshed	12	NP28-29-30R
w12 EW A-B-C	Exposed	Whole body	12	NP31-32-33
w12 EF A-B-C	Exposed	Fleshed limb	12	NP19-20-21
w20 BB A-B-C	Buried	Defleshed	20	NP4-5-6
w20 EW A-B-C	Exposed	Whole body	20	NP1-2-3
w20 EF A-B-C	Exposed	Fleshed limb	20	NP7-8-9
w24 BB A-B-C	Buried	Defleshed	24	NP34-35-36
w24 EW A-B-C	Exposed	Whole body	24	NP37-38-39
w24 EF A-B-C	Exposed	Fleshed limb	24	NP22-23-24
w28 BB A-B-C	Buried	Defleshed	28	NP10-11-12
w28 EW A-B-C	Exposed	Whole body	28	NP13-14-15
w28 EF A-B-C	Exposed	Fleshed limb	28	NP16-17-18

## Data Availability

Normalised and raw abundances for the proteins analysed in this study can be all found in the Supplementary Data S1. The mass spectrometry proteomic data have been deposited to the ProteomeXchange Consortium via the PRIDE [68] partner repository with the dataset identifier PXD026042.

## Supplementary Materials

The following are available online at https://www.mdpi.com/article/10.3390/biology10060460/s1: Data S1: Protein exports for normalized and raw abundances of the samples grouped by: “Fleshed vs. defleshed vs. whole”; “Fleshed vs. defleshed”; “Fleshed vs. whole”; “Defleshed vs. whole”; “Buried vs. exposed”; and “PMI”. Figure S1: Monthly temperatures collected at the HuddersFIELD site and average monthly rainfall at the HuddersFIELD site during the field experiment.
