# Supplementary material for: Bone Diagenesis in Short Timescales: Insights from an Exploratory Proteomic Analysis
Source: Biology (Basel). 2021 May 23;10(6):460. doi: 10.3390/biology10060460 (PMC8224596; doi:10.3390/biology10060460)
Supplement: Supplementary file 1 [file biology-10-00460-s001.zip › biology-1209631- Figure S1.pdf]

Supplementary Material

Bone Diagenesis in Short Timescales: Insights from an Exploratory Proteomic Analysis

[Contents]:

Fig. S1. A) Monthly temperatures collected at the HuddersFIELD site and **B)** average monthly rainfall at the HuddersFIELD site during the field experiment
Data S1. Progenesis protein exported tables with relative abundances for proteins used for PCA plots and ANOVA *p* and *q* values.

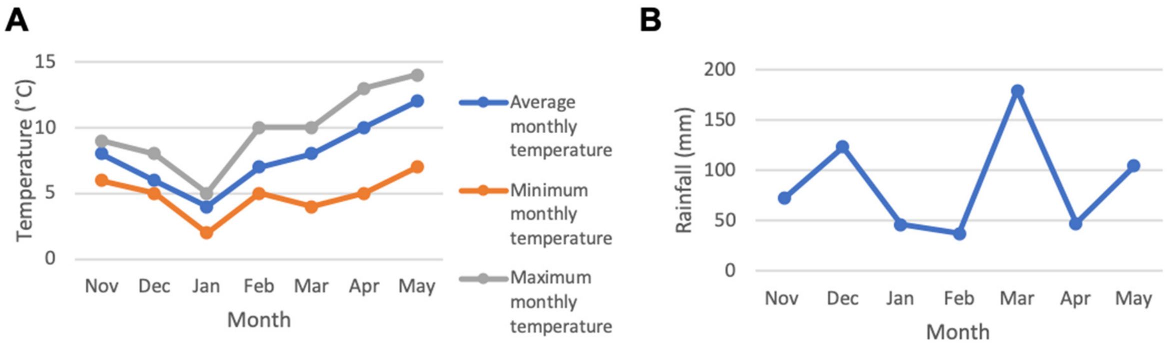

Figure S-1. A) Monthly temperatures collected at the HuddersFIELD site and **B)** average monthly rainfall at the HuddersFIELD site during the field experiment.
